# Video-Assisted Instruction Versus Simulation-Based Mastery Learning for Lumbar Puncture Skill Development in Undergraduates: A Randomized Study

**DOI:** 10.7759/cureus.99178

**Published:** 2025-12-14

**Authors:** Archana Bhat, Vijay Sundarsingh, Manoj Kumar, Lulu Sherif Mahmood

**Affiliations:** 1 Department of Internal Medicine, Father Muller Medical College, Mangalore, IND; 2 Department of Critical Care Medicine, Father Muller Medical College, Mangalore, IND; 3 Department of Anesthesiology, Father Muller Medical College, Mangalore, IND

**Keywords:** lumbar puncture training, medical education, objective structured assessment of technical skills (osats), procedural skill retention, simulation-based mastery learning, technical skills assessment, undergraduate medical students, video-assisted instruction

## Abstract

Background

Lumbar puncture (LP) is a technically demanding procedure fundamental for diagnosis and treatment in multiple specialties. Undergraduate medical training often provides limited opportunities for deliberate practice. Video-assisted instruction and simulation-based mastery learning (SBML) have emerged as promising educational strategies. However, direct comparisons of these modalities for LP training remain scarce.

Methods

This randomized study included 30 final-year MBBS students assigned equally to video-assisted instruction or SBML groups. Both groups received identical learning objectives and practiced LP on simulators. Knowledge was assessed before and after training using a validated questionnaire, while procedural competence was evaluated immediately and at two weeks using the objective structured assessment of technical skills (OSATS). Data were analyzed using mixed-design analysis of variance (ANOVA) and paired t-tests.

Results

The video-assisted group demonstrated significantly higher immediate OSATS scores (mean ± SD: 12.73 ± 2.12) compared to the SBML group (10.47 ± 1.81; p = .004, Cohen’s d = 1.05). At the two-week follow-up, SBML scores improved (11.93 ± 1.28), whereas video-assisted scores declined (11.53 ± 1.64), resulting in a significant group × time interaction (F(1,28) = 8.82, p = 0.004). Both groups achieved significant knowledge gains (mean difference = 1.17; p = 0.017). The retention index favored SBML (114%) compared with video-assisted instruction (91%).

Conclusion

Video-assisted instruction accelerates early acquisition of LP procedural skills, while SBML promotes superior long-term retention. Incorporating both strategies into undergraduate curricula may optimize procedural performance and durability. Such integration has the potential to improve competency development and, ultimately, patient care.

## Introduction

Developing advanced clinical competence is a central goal of contemporary medical education, particularly as curricula require mastery of increasingly complex procedures. The lumbar puncture (LP) remains a core yet technically demanding skill, vital for both diagnostic and therapeutic purposes across multiple specialties. Despite its significance, undergraduate LP training frequently exposes substantial learning gaps. The traditional apprenticeship approach, summarised by the adage “see one, do one, teach one,” has been criticized for inconsistent supervision, insufficient deliberate practice, and variable feedback, all of which can compromise skill acquisition and patient safety [1,2].

Simulation-based education offers a structured and risk-free environment for developing procedural skills. Evidence shows that it enhances competence, builds confidence, and alleviates procedural anxiety [3-6]. Within this domain, simulation-based mastery learning (SBML) is regarded as a gold-standard approach, combining high-fidelity simulation with clearly defined performance standards, deliberate practice, and feedback to ensure all learners meet a minimum competence threshold [7-9]. SBML has been shown to accelerate skill acquisition, promote long-term retention, and reduce performance variability [7,9,10].

Parallel to this, video-assisted instruction has gained prominence for providing accessible, standardised, and repeatable demonstrations [11-13]. While effective for rapid familiarisation, such videos lack the interactive feedback and hands-on engagement required for mastering complex psychomotor skills. Both SBML and video-based approaches offer proven benefits, yet direct comparisons of their effectiveness in LP training, particularly regarding short-term skill retention and theoretical underpinnings, remain limited.

Our study draws on Fitts and Posner’s three-stage model of motor learning (cognitive, associative, and autonomous stages), Ericsson’s deliberate practice framework (structured, goal-oriented practice with feedback), and the cognitive load theory (reducing extraneous mental effort in instructional design) [14-16]. SBML exemplifies the principles of deliberate practice, while video-assisted learning may help lower cognitive load and support schema development for complex procedures.

Although both SBML and video-assisted instruction have established roles in procedural skills training, notable gaps persist in the literature, especially for LP education in undergraduate medical students. Existing research typically examines each method separately, leaving limited evidence from direct comparisons that explore how these approaches differ in supporting immediate skill acquisition and short-term retention [6,17]. This highlights a clear problem: educators lack guidance on which modality better enhances early performance versus sustained competency in LP. To address this, the present study compares SBML and video-based instruction across three key outcomes: (1) improvement in cognitive knowledge, (2) immediate procedural performance measured using the objective structured assessment of technical skills (OSATS) [18], and (3) retention of skills at two weeks. Evaluating these outcomes might offer meaningful insights to inform and strengthen undergraduate procedural skills curricula and align with earlier calls for direct comparative evidence in LP training methodologies.

## Materials and methods

Study design and setting

A single-center, prospective interventional study was conducted at the Simulation Laboratory of Father Muller Medical College, Mangalore, India, following approval from the Institutional Ethics Committee (Ref: FMIEC/CCM/511/2023). The study was designed to compare the immediate and short-term effectiveness of SBML and video-assisted instruction for teaching LP skills to undergraduate medical students.

Participants

Thirty final-year MBBS students were recruited using convenience sampling. Eligibility criteria included willingness to participate and availability during the study timeline. Students with prior exposure to LP training (such as completion of anaesthesiology postings) were excluded to minimize baseline skill variability. All participants provided informed written consent before enrolment. 

Randomization and study flow

**Figure 1:**
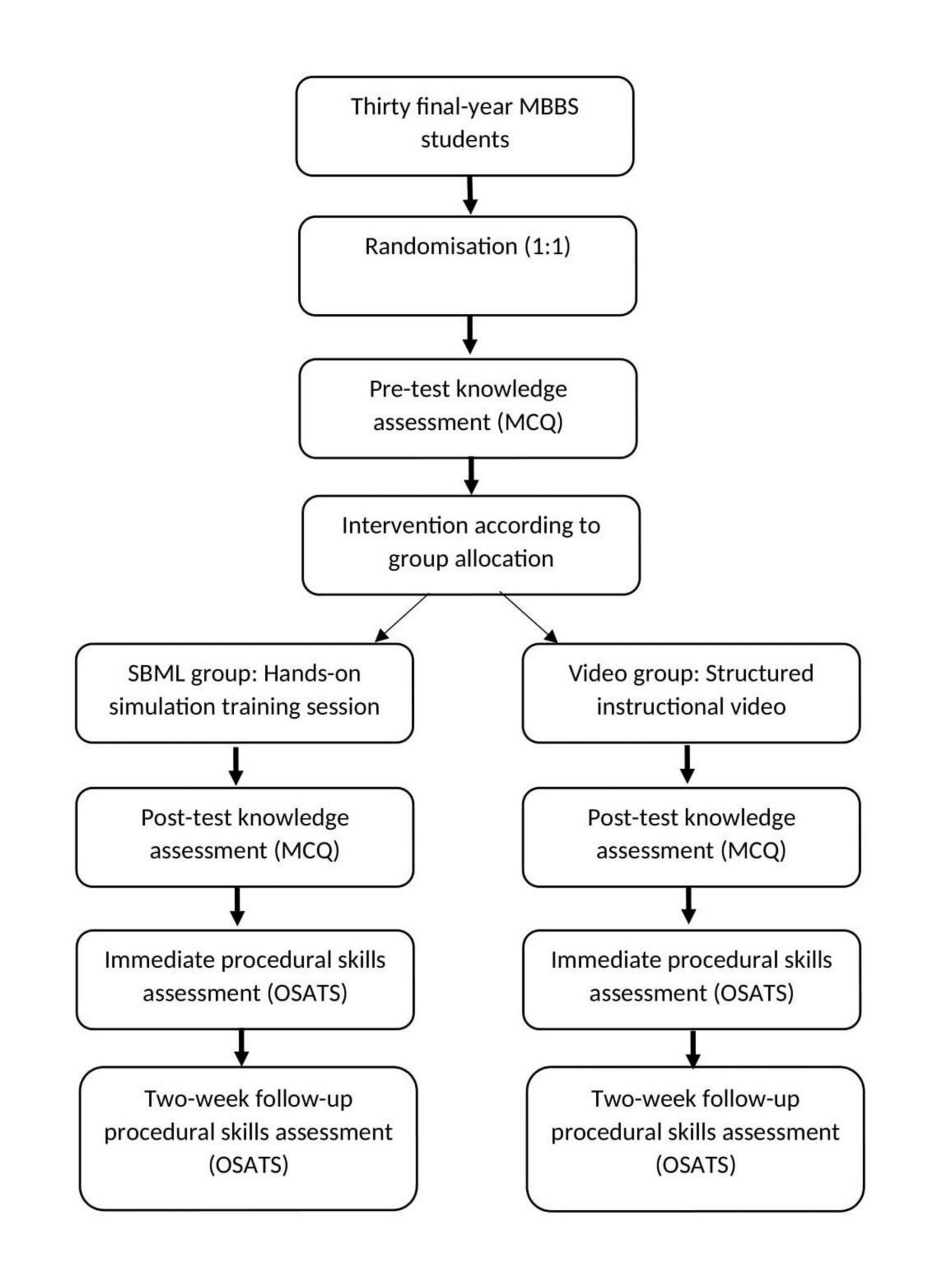
Flow diagram of the study process MBBS: Bachelor of Medicine, Bachelor of Surgery; MCQ: multiple-choice questions; SBML: simulation-based mastery learning; OSATS: objective structured assessment of technical skills

Participants were randomized in a 1:1 allocation to the SBML or video-assisted instruction group. Randomization was accomplished by having an independent staff member draw participant names sequentially from identical slips in a container and alternately assign students to either group, ensuring an unbiased allocation (n = 15 per group). The flow of participants through each stage of the study is illustrated in the study flow diagram (Figure 1).

Educational Interventions

Both instructional methods adhered to identical learning objectives and were delivered on the same day, in separate rooms, to prevent group contamination.

Video-assisted instruction: Participants viewed a 20-minute-long structured instructional LP exemplar video from the NHS Lothian Mastery Programme, which provides detailed, stepwise audiovisual guidance on performing LP procedures [19]. This video offers a standardized and replicable visual demonstration consistent with mastery learning principles. The video is publicly accessible and available for educational purposes.

SBML: Participants engaged in a 45-minute hands-on training session using the Kyoto Kagaku Lumbar Puncture Simulator II (model M43B), an anatomically realistic task trainer supporting practice of all critical LP procedural steps. Training included deliberate practice and formative feedback to meet defined competency standards. 

Assessors and blinding

Two senior professors with >10 years’ experience in simulation-based teaching served as evaluators. Both had prior training in objective skills assessment. To minimize bias, each assessor conducted OSATS evaluations at only one time point (immediate or two-week follow-up) and was fully blinded to participants’ group allocation and type of instruction received. Prior to data collection, the two assessors conducted a brief calibration session in which they jointly reviewed OSATS scoring criteria and discussed common scoring scenarios to enhance consistency in interpretation. No formal inter-rater reliability testing was performed.

Outcome measures

Knowledge Assessment

A validated 10-item multiple-choice questionnaire was used to assess cognitive knowledge of LP. The questionnaire was administered both before and immediately after the intervention. Each item carried one mark, giving a total score of 10 (questionnaire used for knowledge assessment has been provided in Appendix 1).

Procedural Skills Assessment

The OSATS checklist, specifically adapted for LP, was used to evaluate sequential procedural steps (scored as correct/incorrect) at two intervals: immediately after training and at two-week follow-up. The OSATS checklist used in this study was adapted from the OSATS-LP tool developed by Iyer et al. [18]. This checklist was obtained from an open-access publication, permitting its use without the need for additional permissions. To adapt the original neonatal OSATS-LP tool for adult LP, item wording and sequence were modified to reflect the anatomical landmarks, procedural nuances, and equipment relevant to adult patients. The adapted checklist underwent content validation by two anesthesiology faculty members experienced in LP training, who reviewed each item for relevance, clarity, and completeness. The final validated checklist is included in the Appendix Table 7.

Statistical analysis

Data were analysed using IBM SPSS Statistics for Windows, Version 23 (Released 2015; IBM Corp., Armonk, New York, United States), with statistical significance set at p < 0.05. Descriptive statistics (mean ± SD) are reported alongside inferential results. Paired t-tests were used to compare pre- and post-intervention knowledge scores within each group and to compare immediate and two-week OSATS scores within groups. Independent t-tests were applied for between-group comparisons at each time point. A two-way repeated measures analysis of variance (ANOVA) was conducted to assess the interaction between training method (SBML vs video) and time (immediate vs two weeks) on OSATS scores. Effect sizes (Cohen’s d) were calculated for relevant comparisons. Retention and skill decay indices were computed for each group. 

## Results

A total of 30 final year MBBS students participated in the study and were randomly assigned to either the SBML group (n = 15) or the video-assisted instruction group (n = 15). All participants completed the pre- and post-intervention knowledge assessments, as well as a procedural skills evaluation using the OSATS checklist, which was conducted immediately after training and at the two-week follow-up.

Across all participants, the mean knowledge scores improved from 6.80 ± 2.14 at baseline to 7.97 ± 1.03 post-intervention. This improvement was statistically significant (mean difference = −1.17; 95% CI: −2.12 to −0.22; p = 0.017), with a moderate effect size (Cohen’s d = 0.54). Table 1 shows the comparison of pre- and post-test knowledge scores.

**Table 1:** Comparison of pre- and post-test knowledge scores SD: standard deviation; CI: confidence interval

Test time point	Mean ± SD	Mean difference	t-statistic	p-value	95% CI of difference	Effect size (Cohen’s d)
Post-pre-difference	-1.17 ± 2.52	-1.17	t(29) = -2.53	0.017	(-2.12, -0.22)	0.54

Item-level analysis of the knowledge questionnaire revealed significant post-intervention gains in multiple areas. Notable improvements were observed in identifying anatomical landmarks (p = 0.004), selecting the appropriate needle gauge (p = 0.009), and recognizing the anatomical structures traversed by the LP needle (p < 0.001). Knowledge also increased regarding prevention of post-LP headache (p = 0.007) and contraindications to LP (p = 0.029). In contrast, there was no significant change in self-reported procedural confidence (p = 0.215).

Immediately after the training session, the mean OSATS score in the SBML group was 10.47 ± 1.81, compared with 12.73 ± 2.12 in the video-assisted instruction group. This difference was statistically significant (p = 0.004). At the two-week follow-up assessment, the SBML group achieved a mean score of 11.93 ± 1.28, while the video group scored 11.53 ± 1.64. The between-group difference at this point was not statistically significant (p = 0.463). Table 2 shows the comparison of OSATS scores between the simulation and video groups.

**Table 2:** Comparison of OSATS scores between simulation and video groups OSATS: objective structured assessment of technical skills; p-values from independent-samples t-tests. *p < 0.05

Time point	Simulation (mean ± SD)	Video (mean ± SD)	t-statistic	p-value (between groups)
Immediate post-training	10.47 ± 1.81	12.73 ± 2.12	t(28) = -3.16	0.004*
2 weeks post-training	11.93 ± 1.28	11.53 ± 1.64	t(28) = 0.74	0.463

Immediately after training, both groups demonstrated perfect completion for "informed consent-benefits." Rates of "wash hands" were high in both groups (100% for video, 86.7% for SBML), while performance for "put on sterile gloves" (video: 93.3%; SBML: 100%) and "position of patient" (video: 80.0%; SBML: 73.3%) remained largely comparable. However, the video group outperformed the SBML group in several key steps, showing significantly higher completion for cleaning skin with betadine (93.3% vs 53.3%, p = 0.013), using 1% lignocaine for anesthesia (60.0% vs 13.3%, p = 0.008), and inserting the spinal needle toward the umbilicus (66.7% vs 6.7%, p = 0.001). Similarly, maintaining bevel orientation favored the video group (73.3% vs 33.3%,p = 0.028). For the other procedural steps, group differences were less pronounced, and completion rates remained similar. Table 3 presents a detailed comparison of step-specific procedural performance between the SBML and video-assisted instruction groups at both evaluation points. 

**Table 3:** Comparison of OSATS performance between simulation and video groups at immediate and 2-week assessments OSATS: objective structured assessment of technical skills; SBML: simulation-based mastery learning; NA: not applicable The p-values are from chi-square (a) or Fisher’s exact (b) tests comparing group differences at each time point. Statistical significance set at p < 0.05 and indicated by an asterisk (*). This table presents the percentage of participants who correctly completed each checklist item at immediate post-training and two-week follow-up for video and simulation (SBML) groups (adapted with permission from [18])

Checklist item	Video immediate (%)	Simulation immediate (%)	Test statistic (immediate)	p-value immediate	Video 2 weeks (%)	Simulation 2 weeks (%)	Test statistic (2 weeks)	p-value 2 weeks
1. Informed consent–benefits	100.0	100.0	NA	NA	100.0	100.0	NA	NA
2. Informed consent–risks explained	73.3	53.3	1.290	0.256^a^	100.0	100.0	NA	1.000
3. Wash hands	100.0	86.7	NA	0.143^b^	73.3	100.0	NA	0.032*^b^
4. Position of the patient	80.0	73.3	0.186	0.666^a^	80.0	86.7	0.167	0.683^a^
5. Identify correct anatomical site	86.7	86.7	NA	1.000^b^	86.7	73.3	0.667	0.414^a^
6. Put on sterile gloves	93.3	100.0	NA	0.309^b^	100.0	66.7	NA	0.014*^b^
7. Clean skin with betadine	93.3	53.3	6.169	0.013^a^*	73.3	73.3	NA	1.000^b^
8. Drape the patient	100.0	86.7	NA	0.143^b^	80.0	80.0	NA	1.000^b^
9. Use 1% lignocaine for anesthesia	60.0	13.3	7.033	0.008^a^*	46.7	46.7	NA	1.000^b^
10. Insert the spinal needle toward the umbilicus	66.7	6.7	10.828	0.001^a^*	53.3	53.3	NA	1.000^b^
11. Maintain bevel orientation	73.3	33.3	4.828	0.028*	53.3	53.3	NA	1.000^b^
12. Advance needle with periodic CSF checking	93.3	93.3	NA	1.000^b^	80.0	80.0	NA	1.000^b^
13. Collect CSF in proper tubes	60.0	86.7	2.722	0.099^a^	93.3	86.7	0.370	0.543^a^
14. Remove the needle after replacing the stylet	86.7	93.3	0.370	0.543^a^	46.7	86.7	5.412	0.020^a^*
15. Apply dressing and list tests	86.7	66.7	1.679	0.195^a^	86.7	100.0	NA	0.143^b^

At the two-week follow-up, both groups sustained perfect performance for "informed consent-benefits" and "informed consent-risks." The SBML group showed increased adherence in steps such as washing hands (100% vs 73.3%, p = 0.032) and removing the needle after replacing the stylet (86.7% vs 46.7%, p = 0.02), while the video group excelled in putting on sterile gloves (100% vs 66.7%, p = 0.014). Most other checklist items, including identifying anatomical site, draping the patient, advancing needle with periodic CSF checking, and collecting CSF, were completed at high and similar rates in both groups. Additionally, applying dressing and listing the required tests showed good performance across participants. Overall, aside from the noted differences, both training modalities led to comparable step-wise procedural competency at the two-week assessment.

Within-group analysis of OSATS scores from immediate post-training to the two-week follow-up revealed distinct patterns for the two instructional approaches. In the SBML group, the mean scores increased from 10.47 ± 1.81 to 11.93 ± 1.28, a statistically significant improvement (p = 0.017). In contrast, the video group’s mean scores declined from 12.73 ± 2.12 to 11.53 ± 1.64, a change that approached but did not reach statistical significance (p = 0.094). Table 4 shows the within-group comparison of the OSATS scores between immediate post-training and two-week follow-up.

**Table 4:** Within-group comparison of the OSATS scores between immediate post-training and two-week follow-up (n = 15 per group) OSATS: objective structured assessment of technical skills; period 1: immediate post-training; period 2: two-week follow-up The p-values are from paired-samples t-tests. Statistical significance set at p < .05, indicated by an asterisk (*)

Comparison	Group	Time point(s)	Mean ± SD	t-statistic	p-value
Within simulation group	Simulation	Period 1 vs Period 2	10.47 ± 1.81 vs 11.93 ± 1.28	t(14) = -2.61	0.017*
Within video group	Video	Period 1 vs Period 2	12.73 ± 2.12 vs 11.53 ± 1.64	t(14) = 1.76	0.094

**Figure 2:**
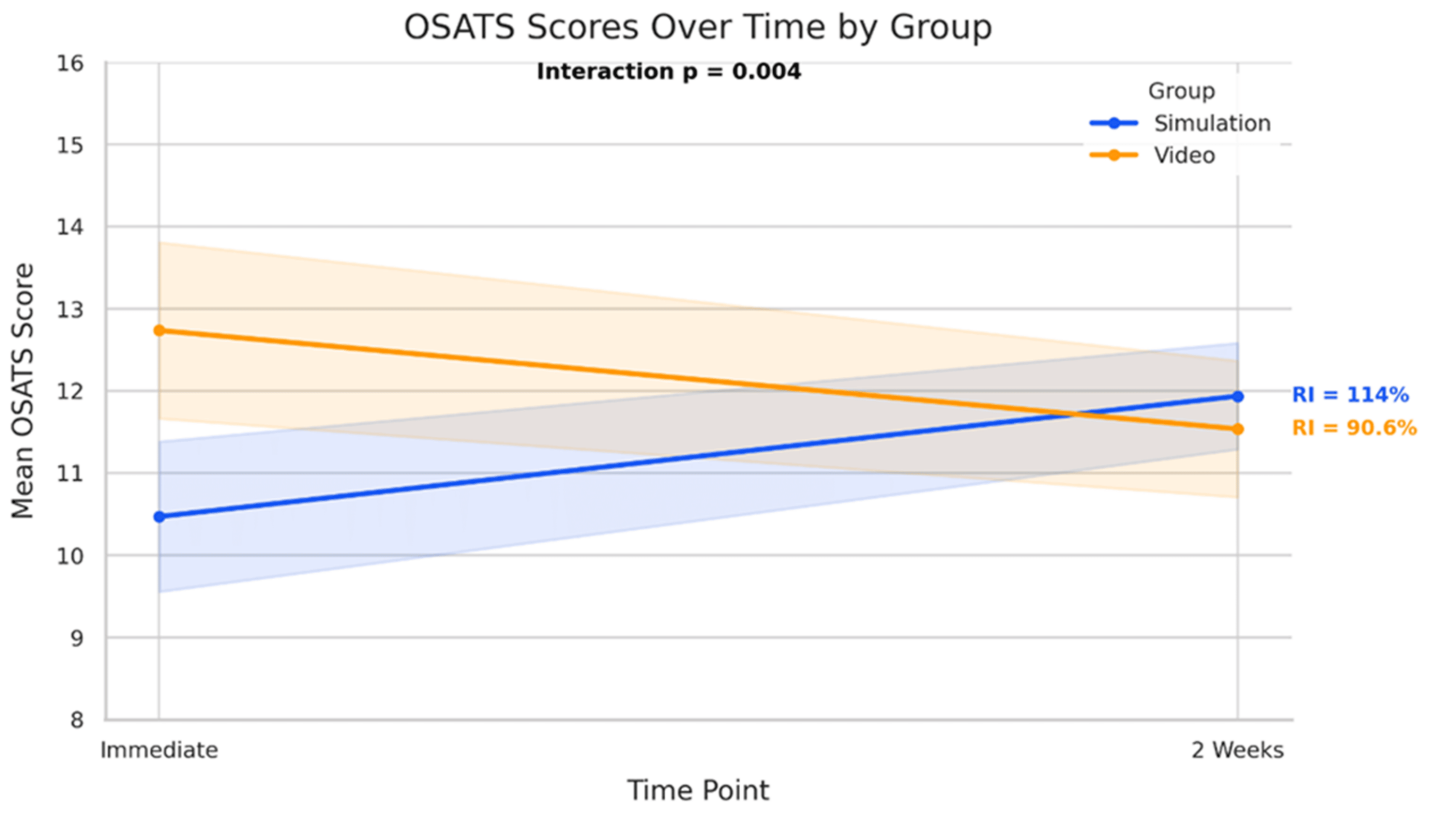
Differential change in the OSATS scores over time between the simulation and video training groups OSATS: objective structured assessment of technical skills; RI: retention index This line chart illustrates the mean OSATS scores for two instructional groups, simulation-based mastery learning (SBML) and video-assisted instruction, across two assessment time points: immediately post-training (OSATS immediate) and at two-week follow-up (OSATS_2week). The simulation group is displayed in blue, while the video group is shown in orange. Shaded areas represent the standard deviation, indicating variability within each group

Statistical indices for skill retention and decay were calculated to assess the changes in procedural competence over time. In the simulation group, the retention index was 114%, with a skill decay index of -1.47. This improvement was statistically significant (paired t-test, p = 0.0009) and accompanied by a large effect size (Cohen’s d = 1.08). In contrast, the video group exhibited a retention index of 90.6% and a skill decay index of +1.20. This decline was also statistically significant (paired t-test, p = 0.036) with a moderate effect size (Cohen’s d = −0.60). The descriptive statistics for OSATS scores, together with retention and skill decay indices, are presented in Table 5. Figure 2 shows the differential change in the OSATS scores over time between the simulation and video training groups.

**Table 5:** Descriptive statistics for the OSATS scores OSATS: objective structured assessment of technical skills; SD: standard deviation

Group	OSATS immediate mean (SD)	OSATS 2-week mean (SD)	Retention index (%)	Skill decay	t-statistic	Paired t-test p-value	Cohen's d
Simulation	10.47 (1.81)	11.91 (1.28)	114.0	-1.47	t(14) = –3.82	0.0019	1.08
Video	12.71 (2.21)	11.53 (1.64)	90.6	+1.20	t(14) = 2.38	0.0360	0.60

A two-way mixed-design ANOVA was performed to examine the effects of training method (simulation-based vs. video-based) and time (immediate post-training vs. two-week follow-up) on the OSATS scores. Results showed a significant interaction between group and time (F(1, 56) = 8.82, p = 0.004). Additionally, the main effect of the training group was statistically significant (F(1, 56) = 4.32, p = 0.042), when considering both time points. However, the main effect of time was not significant (F(1, 56) = 0.09, p = 0.768). The mixed-design ANOVA results for the OSATS scores are summarized in Table 6.

**Table 6:** Mixed-design ANOVA results for OSATS scores OSATS: OSATS: objective structured assessment of technical skills; ANOVA: analysis of variance

Source	Sum of squares	df	F	p-value
Group	13.07	1	4.32	0.042*
Time	0.27	1	0.09	0.768
Group × time	26.67	1	8.82	0.004*
Residual	169.33	56	—	—

## Discussion

Our study compared the effectiveness of video-assisted instruction and SBML in teaching LP skills to final-year MBBS students. The results showed that each instructional approach offered particular strengths at different intervals: video-assisted training led to higher immediate post-training performance, while SBML supported greater skill improvement and retention over the following two weeks. These findings have critical implications for medical education curricula, particularly regarding how procedural skills should be sequenced and reinforced to optimize both skill acquisition and retention.

Immediately after training, students who received video-assisted instruction achieved significantly higher OSATS scores than those trained with SBML. This immediate advantage reflects the effectiveness of high-quality, structured visual content in rapidly conveying stepwise procedural knowledge. Videos engage learners through simultaneous auditory and visual channels, which enhances cognitive encoding of complex information and supports swift skill reproduction, consistent with established theories of multimedia learning [11-13]. The dual sensory input and uninterrupted modelling allow students to internalize the technique efficiently, making video an ideal medium for initial exposure to procedural steps.

However, the longitudinal data revealed a significant interaction effect between group and time (F = 8.82, p = 0.004), indicating that performance trajectories differed considerably across the two groups. By the two-week assessment, the SBML group not only closed the initial performance gap but surpassed the video group, demonstrating superior skill retention. This improvement underscores the well-documented importance of hands-on, deliberate practice with real-time feedback for consolidating procedural memory and psychomotor competence [20]. Unlike declarative knowledge gained from video, durable mastery relies on repeated physical engagement with the task, error correction, and refinement of motor sequences facilitated by SBML’s immersive environment. These findings align with prior research emphasizing simulation’s role in maintaining competence beyond immediate training [20,21].

Item-level analysis using the OSATS highlighted distinct modality-specific advantages. The video-assisted instruction group demonstrated superior early acquisition of tasks characterized by visual complexity but relatively straightforward motor execution, such as needle trajectory, administration of local anesthetic, and bevel orientation. These components align well with guided visual modelling and cognitive rehearsal facilitated by video learning. In contrast, participants in the SBML group excelled in activities requiring advanced psychomotor skills, including precise needle withdrawal and meticulous hand hygiene observed at the two-week follow-up. These tasks demand refined motor control, tactile feedback, and procedural fluency, best developed through deliberate hands-on practice. This divergence in skill domains underscores the complementary nature of the two instructional approaches and supports combining them to optimize both initial skill acquisition and long-term proficiency [20,22].

Both groups demonstrated significant improvements in knowledge related to LP anatomy, needle gauge selection, and the anatomical layers traversed during the procedure. However, self-reported confidence to perform LP under supervision did not show a statistically significant increase following the training. This dissociation between competence and confidence has been documented in prior research [23,24] and may be attributable to the limited duration of the training interventions. It is also important to note that although both groups improved in knowledge and technical performance, students’ confidence to perform the procedure under supervision did not significantly increase. This distinction reinforces that competence and self-efficacy do not always develop in parallel, particularly after brief training sessions. Additionally, our findings reflect only short-term (two-week) retention, and the durability of these skills over longer periods remains unknown. The initial higher OSATS scores in the video group should also be interpreted cautiously, as this early advantage may be partly influenced by cognitive priming, where recent visual exposure enhances immediate recall of procedural steps rather than true psychomotor mastery. Developing procedural confidence often requires prolonged or repeated simulation sessions that combine experiential learning with practical, real-world application [20,25]. These findings highlight that while concise training interventions effectively enhance technical knowledge and skills, additional curricular components are essential to build and sustain professional confidence in performing invasive procedures.

From a theoretical perspective, our findings suggest that optimal LP training should leverage the complementary strengths of each modality across Fitts and Posner's learning stages [15]. Video instruction efficiently addresses cognitive stage requirements by reducing extraneous cognitive load and supporting initial schema development [16]. Subsequent SBML training then facilitates progression through associative and autonomous stages via deliberate practice principles, incorporating the goal-directed, feedback-rich experiences necessary for procedural mastery [14]. This staged approach maximizes both immediate learning efficiency and long-term skill retention.

Our results advocate for a blended educational strategy for LP training in undergraduate curricula. An initial video session can effectively establish cognitive scaffolding by enabling learners to visualize and mentally rehearse the procedural steps. SBML sessions should rapidly follow this to consolidate motor skills through active, feedback-rich practice that enhances skill retention and safety-critical behaviours. Such a sequence aligns with the staged competency framework articulated by Manthey and Fitch, emphasizing progressive skill acquisition from knowledge to applied mastery [26].

Furthermore, the two-week gain observed in the SBML group suggests that simulation training may encourage post-session mental rehearsal and retrieval practice independently, thereby reinforcing procedural memory without the need for formal refresher sessions. Curriculum planners should therefore consider scheduling video and simulation components on the same day or close together to optimize synergistic learning.

Future research should evaluate the generalizability of this blended approach across different clinical procedures and learner populations through large-scale, multi-center trials. Investigations into the optimal timing and frequency of refresher or booster sessions could clarify how to sustain skill competence most effectively over time. Qualitative studies exploring learner perceptions of video versus simulation modalities may provide insight into motivational and affective factors influencing engagement and retention. Moreover, longitudinal designs examining the translation of simulated competence into clinical performance and patient outcomes would be valuable.

The study directly compared two widely used instructional modalities (video-assisted instruction and SBML) in a randomized design, providing robust evidence on their distinct advantages in procedural skill acquisition and retention. Secondly, the comprehensive assessment included both knowledge and objective procedural skills (OSATS) measured immediately post-training and at two weeks, allowing evaluation of both short-term gain and skill retention over time. Finally, the use of objective, validated assessment tools (OSATS checklist) and detailed item-level analysis enabled a comprehensive understanding of modality-specific strengths in visually complex versus psychomotor-intensive tasks 

Limitations

This study has certain limitations that warrant consideration. Although the initial sample of 30 participants was selected through convenience sampling, post hoc analysis indicated that this size exceeded the minimum requirement to detect the observed between-group difference in OSATS change scores (calculated as 7 participants per group for α = 0.05, 80% power). Nevertheless, the relatively small cohort may still limit the detection of subtle effects and restrict the generalizability of the findings to other learner populations, institutions, or healthcare settings.

All participants came from a single medical college with its own curriculum and local context, so repeating the study in different cultural and educational settings is needed to see if the results hold. We also studied only one procedure, LP, so the findings may not apply to skills that require different types of cognitive or motor abilities. The two teaching methods also differed in duration and level of interaction: the video session lasted 20 minutes, while the SBML session lasted 45 minutes, which may have contributed to the early performance differences seen between groups. 

In terms of validity, we used a validated OSATS checklist to assess procedural competence, which supports the internal validity of our findings. However, all assessments took place in a simulated environment, not in real clinical practice. This means our results may not fully reflect how students perform with real patients, where factors such as patient variability, environmental challenges, and stress could affect outcomes. In addition, the same faculty delivered the training and assessed performance, which could introduce subtle assessor bias, even though the assessors were blinded to group allocation. We did not employ stratified randomisation, which may have resulted in imbalances in baseline characteristics such as prior procedural exposure or academic performance.

For outcomes, we measured both cognitive knowledge and procedural skill objectively rather than relying on self-reported ability, which aligns with best practices for performance-based assessment. However, we did not measure clinical outcomes or patient impact, so we cannot conclude the real-world effectiveness of the training. Self-reported confidence did not significantly improve, showing that feeling confident and being competent are not always the same. Finally, our two-week follow-up period was too short to evaluate long-term skill retention or the benefit of refresher training, and we did not explore other factors such as motivation or anxiety, which could also play a role in maintaining skills over time.

## Conclusions

Our study shows that both video-assisted instruction and SBML have unique strengths in teaching LP skills to undergraduate medical students. Video-based training helps learners pick up visually guided steps in the early stages, while SBML supports continued improvement and better retention of hands-on psychomotor skills. A curriculum that combines these methods in a planned sequence can make the most of both approaches, boosting immediate performance, maintaining skills over time, and ultimately improving patient safety and care quality.

## Appendices

Appendix 1: questionnaire (Google Form) for knowledge assessment

 1. The favored position of patients in adults for lumbar puncture 

A) Universal flexion of the body 

B) Extension of the back 

C) Standing 

D) Sitting 

2.  The anatomical landmark iliac crest corresponds to 

 a) L1-L2

 B) L2-L3

 C) L3-L4

 D) L4-L5

3. Needle gauge used for adult LP procedure is all, except 

A) 18 G

B) 20 G

C) 21G

D) 25 G

4. Indications for LP include all except

A) Raised intracranial pressure 

B)  Meningitis

C) Intrathecal methotrexate 

D) Normal pressure hydrocephalus 

5. The site of LP needle insertion is all except 

A) L2-L3

B) L1-L2

C) L3-L4

D) L4-L5

6. Structures that the LP needle passes in order are

A) Supraspinous ligament, ligamentum flavum, subarachnoid space, dura mater, epidural space

B) Ligamentum flavum, epidural space, arachnoid mater, duramater, subarachnoid space

C) Supraspinous ligament, epidural, duramater, arachnoid, subarachnoid space

D) Supraspinous ligament, arachnoid mater, dura mater, epidural space

7. Contraindications for LP are all except

A)   Papilledema

B)   CNS meningitis 

C)   Posterior fossa tumor

D)   Cerebellar bleed

8. Confidence about the procedure to be done under supervision 

A) Yes 

B) No

9. Post-LP headache may be reduced by all except 

A) Paracetamol 

B) Supine posture 

C) Blood patch 

D) Sitting 

10. Samples to be sent for CSF analysis are all except 

A) Cell count 

B) Biochemistry 

C) Oligoclonal band 

D) Creatinine

**Table 7:** Checklist for OSATS OSATS: objective structured assessment of technical skills; CSF: cerebrospinal fluid

Steps done	Performed	Not performed
1. Consent taken		
2. Indication and procedure explained, side effects told, and keep CSF kit ready		
3. Positioning the patient with knowledge of the correct anatomic position		
4. Hand hygiene		
5. Put on sterile gloves		
6. Clean skin with aseptic solution		
7. Drape the patient		
8. Use 1% lignocaine to give local anesthesia to skin and deeper		
9. Insert Spinal Needle Directed Towards Umbilicus In The Midline		
10. Slowly Advance Needle With Periodic Checking For CSF		
11. Collect CSF Using Manometer Or Tubes And List The Tests To Be Sent		
12. Remove Needle After The Stylet Is Replaced		
13. Disposal Of Waste And Sharps Appropriately		
14. Apply Simple Dressing To The Insertion Site		
15. Document The Procedure And Post Procedure Care		
